# S3 Dorsal Root Ganglion/Nerve Root Stimulation for Refractory Postsurgical Perineal Pain: Technical Aspects of Anchorless Sacral Transforaminal Lead Placement

**DOI:** 10.1155/2016/8926578

**Published:** 2016-03-30

**Authors:** X. Zuidema, J. Breel, F. Wille

**Affiliations:** ^1^Department of Anaesthesiology and Pain Medicine, Diakonessenhuis Utrecht/Zeist, Internal Post Box 53, P.O. Box 1002, 3700 BA, Zeist, Netherlands; ^2^Department of Anaesthesiology and Pain Medicine, Academic Medical Centre, P.O. Box 22660, 1100 DD Amsterdam, Netherlands

## Abstract

Chronic perineal pain limits patients in physical and sexual activities, leading to social and psychological distress. In most cases, this pain develops after surgery in the urogenital area or as a consequence of trauma. Neuromodulation is one of the options in chronic postsurgical perineal pain treatment. We present a case of refractory perineal pain after right sided surgical resection of a Bartholin's cyst which was treated with third sacral nerve root/dorsal root ganglion stimulation using the transforaminal approach. We describe a new anchorless lead placement technique using a unique curved lead delivery sheath. We postulate that this new posterior foraminal technique of lead placement is simple, safe, and reversible and may lower the occurrence of lead related complications.

## 1. Introduction

Chronic perineal pain is a disabling and invalidating syndrome. Patients are often limited in physical and sexual activities, leading to social and psychological distress. Chronic perineal pain may occur after surgical intervention or trauma in the perineal area or may be caused by inflammation (e.g., prostatitis) or pudendal nerve entrapment. Chronic postsurgical perineal pain (CPSPP) is defined as pain arising after a surgical procedure, present for at least 2 months, with no organic (active cancer or chronic infection) or preexisting cause [1].

The incidence of CPSPP is reported to be up to 38% [2, 3]. Conventional treatment for CPSPP consists of antineuropathic analgesics and minimal invasive pain treatments such as pudendal nerve corticoid infiltration, pulsed radio frequent (PRF) lesions of nerve root S3, or chemical distal sympathectomy [4].

The perineum is innervated by the somatosensory nervous system via sacral nerve roots (pudendal nerve) and the thoracolumbar sympathetic nervous system. Regulatory systems for nociceptive input are present at all levels of the nervous system. The target for perineal pain treatment by neurostimulation may therefore not be a single level but a multilevel approach [5]. Evidence for treatment of refractory CPSPP using invasive neurostimulation techniques is growing; these include peripheral (pudendal) nerve stimulation, sacral nerve root (SNR) stimulation, dorsal root ganglion (DRG) stimulation, spinal cord stimulation (SCS), and motor cortex stimulation [6–9].

We present a patient suffering from chronic postsurgical perineal pain and treated with third sacral nerve root/dorsal root ganglion stimulation (3rd SNR/DRG) using the transforaminal approach. This case report describes the procedural aspects of anchorless sacral transforaminal lead placement.

## 2. Case Description

A 58-year-old woman was referred to our pain clinic with refractory perineal pain after right sided surgical resection of a Bartholin's cyst. She reported suffering from perineal pain of an allodynic and hyperalgesic nature for more than 6 years. Further comorbidity included non-insulin dependent diabetes mellitus, hypertension, hypercholesterolemia, and a cerebrovascular accident with little functional impairment. Antineuropathic analgesics (tramadol, oxycodone, pregabalin, and amitriptyline) as well as minimal invasive techniques (right sided pulsed radio frequent lesion of nerve root S3, chemical neurolesion of Impar's ganglion, and hypogastric plexus block) for neuropathic pain treatment did not result in structural pain relief. Over the last 2 years, her pain had steadily become more intense, resulting in functional, psychological, sexual, and social dysfunction.

After discussing neuromodulation options, the patient consented to a stimulation trial of the 3rd SNR/DRG. Prior to implantation, a retrograde transforaminal paraesthesia mapping (RTPM) procedure of the right sided S3 nerve root was performed (this technique was described earlier [10]), producing 100% coverage of the painful area.

## 3. Procedure Aspects

The lead placement procedure was performed under conscious sedation (remifentanil) and field infiltration anesthesia (lidocaine 2%). Under fluoroscopic guidance, a quadripolar stimulation lead (Spinal Modulation, Inc., Menlo Park, CA, USA) was placed over the DRG and NR of S3 on the right side, using a unique curved delivery sheath. In order to prevent lead displacement, fixation of the lead was obtained by threading three lead loops into the sacral epidural space.

This was achieved by retraction of the delivery sheath in the sacral epidural space, together with readjustment of the needle bevel (turned to a different angle), and sheath curve (more cranially). The lead was further delivered up into the epidural space without displacement of the initial tip placement.

**Figure 1:**
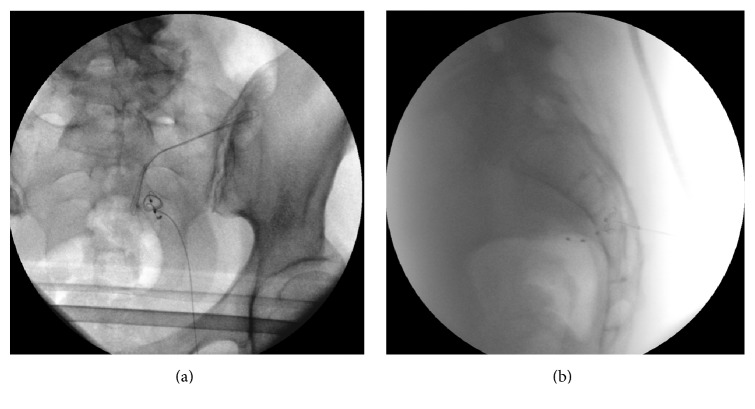
(a) AP view S3 lead placement with epidural loops. (b) Lateral view S3 lead placement with epidural loops.

The lead has a natural tendency to fold due to its flexibility, so we were able to make fixation loops of the lead in the sacral epidural space (Figures 1(a) and 1(b)).

No further anchoring was necessary to secure the lead. After perioperative confirmation of optimal paraesthesia coverage in the painful area, coupled with a 50% reduction in pain, an implantable pulse generator (IPG, Spinal Modulation Axium) was implanted in the right buttock in the same session.

## 4. Results

The patient experienced an 80% pain relief in the 2 weeks after implantation. The pain intensity measured by the visual analogical scale (VAS) dropped from 90 to 10.

Time from incision to lead placement was 3 minutes, stimulation duration was 5 minutes, total surgical time was 35 minutes, and total procedure time was 52 minutes.

Programming parameters are shown in Table 1.

Neither lead nor IPG related complications were observed.

## 5. Discussion

Evidence for the efficacy of neuromodulation in refractory perineal pain is growing. A few case reports describe good pain relief obtained by stimulating the medullary conus, using 16-polar plate electrodes [7, 11, 12]. A major drawback to these leads, however, is that a laminectomy has to be performed in order to place the electrode, the technique is irreversible, and an MRI cannot be performed after placement.

3rd SNR/DRG stimulation is not a new technique in the treatment of perineal pain syndrome. Alo et al. and De Andres et al. described patients with perineal pain experiencing excellent pain relief using the retrograde lead placement technique [13, 14]. This technique is quite difficult to perform and may increase procedure time whilst optimal paraesthesia coverage in the OR is sought. We used the easier posterior transforaminal approach to introduce the lead over the NR and the DRG. Lavano et al. also used the latter technique and reported good pain relief and improvement in quality of life. A relatively high risk of complication mainly due to lead related issues (fracture and displacement), however, was observed [15]. To overcome the problem of lead displacement, implanters may choose to use a tined lead (Medtronic Inc.), which is inserted into the S3 foramen. This might reduce the risk of displacement but may lead to nerve damage if the lead was to be explanted (e.g., infection, negative trial period). If explantation was necessary in our case, then the lead could simply be withdrawn (this is also facilitated by the lack of anchor), leaving no further damage to the nerve tissue. We postulate that this new posterior foraminal technique of lead placement is simple, safe, and reversible and may lower the occurrence of lead related complications. As this is a single case study, this hypothesis would have to be tested in a larger study.

## Figures and Tables

**Table 1:** Programming parameters.

Parameter	Initial	Week 2
Stimulation poles	N + −N	+ − N, N
Pulse width (ms)	300	100
Amplitude (pA)	250	250
Frequency (Hz)	20	20
Resistance (Ohm)	185013211311active1313	107010631353active1070
